# The relationship between serum sodium and intracranial pressure when using hypertonic saline to target mild hypernatremia in patients with head trauma

**DOI:** 10.1186/cc11678

**Published:** 2012-10-15

**Authors:** Diana L Wells, Joseph M Swanson, G Christopher Wood, Louis J Magnotti, Bradley A Boucher, Martin A Croce, Charles G Harrison, Michael S Muhlbauer, Timothy C Fabian

**Affiliations:** 1 Department of Pharmacy Practice, Auburn University Harrison School of Pharmacy, 1321 Walker Building, Auburn, AL 36849, USA; 2 Department of Clinical Pharmacy, University of Tennessee Health Science Center, 881 Madison Avenue, Memphis, TN 38163, USA; 3 Department of Surgery, University of Tennessee Health Science Center, 910 Madison Avenue, Memphis, TN 38163, USA; 4 Department of Neurosurgery, University of Tennessee Health Science Center, 427 Johnson Building, Memphis, TN 38163, USA

## Abstract

**Introduction:**

Limited data suggest mild hypernatremia may be related to lower intracranial pressure (ICP) in patients with traumatic brain injury (TBI). The practice at the study center has been to use hypertonic saline (HTS) to generate a targeted serum sodium of 145 to 155 mEq/l in patients with TBI. The purpose of this study was to determine the relationship between serum sodium values and ICP, and to evaluate the acute effect of HTS on ICP.

**Methods:**

A retrospective review of patients who were admitted to the trauma ICU for TBI, had an ICP monitor placed, and received at least one dose of HTS between January 2006 and March 2011 was performed. Data were collected for up to 120 hours after ICP monitor placement. The primary outcome was the relationship between serum sodium and maximum ICP. Secondary outcomes were the relationship between serum sodium and the mean number of daily interventions for ICP control, and the acute effect of HTS on ICP during the 6 hours after each dose. Linear regression was used to analyze the primary outcome. Analysis of variance on ranks and repeated measures analysis of variance were used to evaluate the number of interventions and the acute effect of HTS on ICP, respectively.

**Results:**

Eighty-one patients were enrolled with mean ± standard deviation age of 36 ± 15 years and median Glasgow Coma Scale score of 7 (interquartile range, 4 to 7). A total of 1,230 serum sodium values (range, 118 to174 mEq/l) and 7,483 ICP values (range, 0 to 159 mmHg) were collected. There was no correlation between serum sodium and maximum ICP (*R*^2 ^= 0.0052). The overall mean ± standard deviation number of interventions for elevated ICP per day was 4.2 ± 2.9, 2.9 ± 2.0, and 2.6 ± 2.3 for patients with a mean serum sodium of < 145, 145 to 155, and > 155 mEq/l, respectively (*P *< 0.001). Regarding the acute effect of HTS on ICP, there was no statistical difference in mean ICP compared with baseline during hours 1 through 6 following HTS doses (baseline, 13.7 ± 8.4 mmHg; hour 1, 13.6 ± 8.3 mmHg; hour 2, 13.5 ± 8.8 mmHg; hour 3, 13.3 ± 8.7 mmHg; hour 4, 13.4 ± 8.7 mmHg; hour 5, 13.4 ± 8.3 mmHg; hour 6, 13.5 ± 8.3 mmHg; *P *= 0.84).

**Conclusions:**

Serum sodium concentrations did not correlate with ICP values. These results warrant further evaluation and possible reassessment of sodium goals for ICP management in patients with TBI.

## Introduction

Traumatic brain injury (TBI) is the leading cause of morbidity and mortality in young adults [1]. When treating TBI, optimal intracranial pressure (ICP) control (that is, < 20 mmHg) and an adequate cerebral perfusion pressure of 50 to 70 mmHg are goals associated with lower mortality [2]. While mannitol has long been a mainstay of ICP management, the use of hypertonic saline (HTS) has more recently gained increasing support. This increased usage is likely because of a perceived decrease in adverse effects in comparison with mannitol such as excessive volume loss and rebound ICP elevation [3]. However, the Brain Trauma Foundation guidelines for the management of TBI note that the strength of the current literature does not substantiate a formal recommendation on the use of HTS to lower ICP [2].

Small clinical studies in patients with TBI have suggested that the acute effects of HTS administration on ICP may be equivalent to or in some cases more substantial and longer lasting than mannitol [4,5]. These studies seem to support the use of HTS for acute ICP management in patients with TBI. However, some centers use a somewhat different approach in using HTS to target serum sodium of 145 to 155 mEq/l in the hope of providing a continuous ICP-lowering effect. This is based on limited retrospective data suggesting that targeting a serum sodium concentration of 145 to 155 mEq/l in patients with TBI may correlate with a reduction in ICP [6]. More data on the safety and efficacy of this approach are clearly needed.

At the study institution HTS is administered every 6 hours to target a serum sodium goal of 145 to 155 mEq/l. Our study group hypothesized that targeting serum sodium concentrations of 145 to 155 mEq/l would correlate with lower ICP values. To test this hypothesis, we performed a retrospective review with the following specific aims: to define the relationship between serum sodium concentration and ICP; to determine the relationship between serum sodium concentration and the number of daily interventions for intracranial hypertension in patients with TBI; and to determine the acute change in ICP following a HTS bolus.

## Materials and methods

### Study design and patient population

This retrospective review was approved by the Institutional Review Board of the University of Tennessee Health Science Center and the Regional Medical Center at Memphis. All consecutive patients admitted to the Regional Medical Center at Memphis for TBI between January 2006 and March 2011 who received at least one dose of HTS as documented in the medication profile (Meditech, Medical Information Technology, Westwood, MA, USA) were identified for screening. Those who met the following criteria based on retrospective review of the medical record were enrolled in the study: age ≥ 18 years, treatment with HTS confirmed by medication administration records, and ICP monitor placement. Patients were excluded if their neurologic insult was not due to TBI. Owing to the retrospective nature of the study, all treatment occurred as part of routine patient care, and the requirement for informed consent was waived.

Data from the medical record and a trauma registry (NTRACS, version 3.0; Digital Innovation, Forest Hill, MD, USA) were used to determine patient demographics, Injury Severity Score, daily Glasgow Coma Scale (GCS) score, mechanism of trauma resulting in TBI (for example, fall, motor vehicle collision, assault, pedestrian struck, gunshot wound), type of trauma (blunt vs. penetrating), neurosurgical procedures (for example, ventriculostomy, craniectomy), number and type of medical interventions per day for ICP management (for example, HTS, mannitol, sedation, neuromuscular blockade), hourly ICP, hourly cerebral perfusion pressure, laboratory information (for example, serum creatinine, serum sodium), hospital length of stay, duration of mechanical ventilation, and in-hospital mortality. Data pertaining to ICP management were collected for up to 120 hours after ICP monitor placement for each patient.

### Treatment

Upon admission to the Regional Medical Center at Memphis, patients with severe TBI had ICP monitors (Camino^®^; Integra LifeSciences Corporation, Plainsboro, NJ, USA) placed at the discretion of a neurosurgery attending physician. Owing to the retrospective nature of the study, data pertaining to the location of the ICP monitor in relation to the area of injury were not always available and therefore these data were not recorded. Following the ICU standard of care, ICP and cerebral perfusion pressure data were recorded by nursing staff on an hourly basis. Nurses are trained to evaluate elevated ICP values, and therapy for intracranial hypertension is only implemented if the value is not a spurious result (for example, not due to coughing). No formal protocol is used for the treatment of intracranial hypertension. However, neurosurgeons in the ICU have a standard approach that includes monitoring the serum sodium concentration every 6 hours and using HTS to raise it above 145 mEq/l. Most patients received 150 ml of 3% HTS intravenously over 2 hours; however, patients who received 150 ml of 7.5% HTS intravenously over 2 hours or continuous intravenous infusion of 3% HTS were also reviewed for this study.

### Primary and secondary outcomes

The primary outcome was the relationship between serum sodium and the corresponding maximum ICP. Secondary outcomes included the relationship between the serum sodium and the number of therapeutic interventions for increased ICP management, and the acute effect of HTS boluses on ICP.

### Definitions

Three methods were used to evaluate the primary outcome. First, each serum sodium value was matched with the highest corresponding ICP recorded before the next serum sodium was measured. The highest ICP value was chosen because intracranial hypertension is clinically important and warrants therapy in TBI patients. This matching was repeated for each serum sodium measurement while the ICP monitor was in place. Missing ICP data were replaced with the last recorded ICP measurement. Second, the change in serum sodium was matched with the change in ICP. To determine the change in serum sodium, the value prior to a HTS bolus was subtracted from the serum sodium value following that bolus. The ICPs measured in the same hour as each serum sodium value were used to determine the corresponding change in ICP. These values were chosen because they best represent the ICP that corresponds to the serum sodium. Third, to account for interpatient variability, serum sodium and the corresponding maximum ICP for each individual patient were also determined. Linear regression was used to identify any relationship between serum sodium and ICP for each of the three methods.

To evaluate the secondary outcome of relating serum sodium to the number of interventions for intracranial hypertension, patients were divided into three groups based on their mean daily serum sodium: < 145 mEq/l, 145 to 155 mEq/l, and > 155 mEq/l. The mean number of interventions per day on days 1 to 5 was compared among these three groups. The following treatments were included as an intervention: HTS infusion, mannitol infusion, sedation or analgesia given by continuous infusion, neuromuscular blocker (NMB) infusion, pentobarbital infusion, or acute hyperventilation (partial pressure of carbon dioxide < 35 mmHg). Any single dose of HTS, mannitol, or NMB was counted as one intervention. Continuous infusions of HTS or NMB were counted as one intervention. Acute hyperventilation was documented as an intervention if an arterial blood gas was available. If more than one arterial blood gas was drawn on any given day for a single patient, only one partial pressure of carbon dioxide < 35 mmHg counted as an intervention for that patient. If a patient's serum sodium was not available on a given day, interventions for that day were excluded from the analysis. Neurosurgical interventions were not included in this analysis, because it was not possible to relate the timing of these procedures to the ICP readings, as details were not consistently available.

To evaluate the secondary outcome of the acute effect of HTS on ICP, only HTS boluses were included. The baseline ICP was defined as the measurement immediately prior to a HTS bolus. The baseline ICP was compared with the ICP from each hour following the HTS bolus. Each bolus was classified as having a positive or negative response on the ICP. A positive response was deemed as any reduction in ICP (negative change in ICP) within the first hour following a bolus. A negative response was either no change or an increase in ICP within the first hour. Patients' responses to HTS boluses were also evaluated based on the number of doses that resulted in a positive response or a negative response. Ultimately, patients were placed into one of two categories based on their response to all boluses. Poor responders (*n *= 41) demonstrated a positive response following 0 to 49% of HTS boluses. Good responders (*n *= 31) demonstrated a positive result following 50 to 100% of HTS boluses.

### Statistical analysis

Continuous variables were compared using Student's *t *test for normally distributed variables or the Wilcoxon rank-sum test for non-normally distributed variables. Proportions were compared using the chi-squared test. Linear regression was used to evaluate the relationship between serum sodium levels and corresponding maximum ICP. Linear regression was also used to analyze the relationship between change in serum sodium and the corresponding change in ICP. Analysis of variance on ranks was used to compare the number of daily interventions among the three groups categorized by serum sodium. Multiple pairwise comparisons were made using Dunn's method. Repeated-measures analysis of variance was used to evaluate the acute effect of HTS on ICP over the 6 hours following a bolus. All statistical analyses were performed using SigmaPlot, version 11.0 (Systat Software Inc., San Jose, CA, USA). Data are presented as mean ± standard deviation, median (interquartile range), or proportions as indicated. *P *< 0.05 was considered statistically significant.

## Results

A total of 81 patients with TBI were enrolled into the study. Baseline demographics are presented in Table 1. Demographics were representative of the patient population with TBI commonly seen in the study ICU [7-9]. Sixty-eight patients (84%) received 3% sodium chloride boluses only. The remaining patients received a combination of 3% and 7.5% sodium chloride boluses, and/or continuous infusions of 3% sodium chloride.

**Table 1:** Baseline demographics and clinical characteristics

Characteristic	Value
Age (years)	36 ± 15
Serum creatinine (mg/dl)	1 ± 0.4
Serum sodium (mEq/l)	139 ± 7
Baseline intracranial pressure (mmHg)	19 ± 11.5
Glasgow Coma Scale	7 (4 to 7)
Injury Severity Score	34 (25 to 41)
Male sex	65 (80%)
Caucasian	54 (67%)
Mechanism of injury	
Blunt trauma	77 (95%)
Penetrating trauma	4 (5%)
Traumatic brain injury cause	
Motor vehicle collision	47 (58%)
Fall	17 (21%)
Assault	6 (7%)
Pedestrian struck	6 (7%)
Gunshot wound	5 (6%)
Isolated traumatic brain injury	30 (37%)
Reactive pupils	52 (64%)
Midline shift	38 (47%)
Craniotomy/craniectomy	46 (58%)
Ventriculostomy	31 (38%)
In-hospital mortality	33 (41%)
Length of stay (days)	
ICU	20 ± 17
Hospital	23 ± 21
Duration of mechanical ventilation (days)	13 ± 10

Data presented as mean *± *standard deviation, median (interquartile range), or number (%) of patients. *n *= 81.

**Figure 1:**
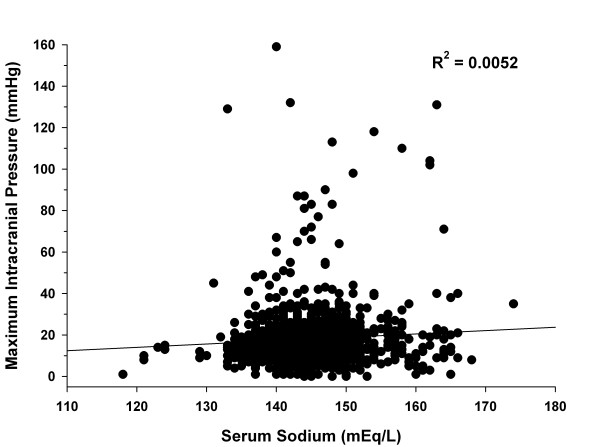
**Scatter plot for correlation between serum sodium and maximum intracranial pressure for all patients**.

**Figure 2:**
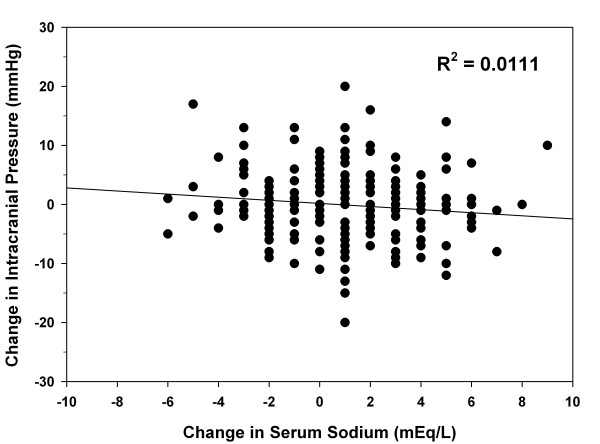
**Scatter plot for correlation between change in serum sodium and change in intracranial pressure for bolus doses of 3% sodium chloride**.

A total of 1,230 serum sodium values (sodium range, 118 to 174 mEq/l) and 7,483 ICP values (ICP range, 0 to 159 mmHg) were collected. Figure 1 shows patients' serum sodium concentrations and their corresponding maximum ICP. Overall, no significant correlation was seen (*R*^2 ^= 0.0052). There was no significant correlation between serum sodium and maximum ICP for subgroups of patients with a baseline ICP < 25 mmHg or ≥ 25 mmHg (*R*^2 ^= 0.0003 and 0.0134, respectively). Furthermore, no correlation between these variables was seen when patients were grouped by GCS score (GCS 3 to 8, *R*^2 ^= 0.0045; GCS 9 to 12, *R*^2 ^= 0.0010; GCS 13 to 15, *R*^2 ^= 0.3084). The association between the change in serum sodium concentration and change in ICP is depicted in Figure 2. The correlation between these two variables was poor (*R*^2 ^= 0.0111). Finally, correlations of serum sodium and ICP analyzed for each patient were poor, as indicated by the number of patients in each *R*^2 ^quartile (Table 2). These results were similar for the entire patient group or when analyzed by subgroups that might affect ICP control (Table 2).

**Table 2:** Individual patient serum sodium and intracranial pressure correlations

**Patient group**	**Patients in each *R*^2 ^quartile**			
	
	**< 0.25**	**0.26 to 0.5**	**0.51 to 0.75**	**> 0.75**
All patients	63 (81%)	9 (11%)	6 (8%)	0 (0%)
Those with craniotomy/craniectomy	33 (77%)	6 (14%)	4 (9%)	0 (0%)
Those with ventriculostomy	28 (90%)	2 (7%)	1 (3%)	0 (0%)
Those with continuous infusion hypertonic saline	10 (100%)	0 (0%)	0 (0%)	0 (0%)
Those without ventriculostomy, craniotomy/craniectomy, or continuous infusion hypertonic saline	19 (90%)	2 (10%)	0 (0%)	0 (0%)

Data presented as *n *(%). Each serum sodium measurement was matched to the corresponding intracranial pressure to calculate individual *R*^2 ^values for every patient. Patients were divided into different groups based on whether or not they received various treatments and/or procedures during the study period (for example, craniectomy, ventriculostomy, continuous infusion hypertonic saline). The number of patients per group falling into each *R*^2 ^quartile is provided.

The mean number of daily interventions per patient based on serum sodium concentration is depicted in Table 3. Interventions included administration of 388 HTS doses, 133 mannitol doses, 54 pentobarbital doses, 332 sedation doses, 198 NMB doses, and 118 acute hyperventilation procedures. There was a statistically significant difference in the total number of interventions over the 5 days for patients with mean serum sodium values < 145 mEq/l when compared with either patients with serum sodium values of 145 to 155 mEq/l or those with values > 155 mEq/l (*P *< 0.05 for each) (Table 3). Statistically significant differences were noted on days 2, 3, and 5, but not for days 1 or 4 (Table 3). When HTS boluses were excluded from the intervention analyses, there was no difference in the number of interventions over the 5 days among the sodium groups < 145 mEq/l, 145 to 155 mEq/l, or > 155 mEq/l (*P *= 0.934), or at any other point during the study period (day 1, *P *= 0.415; day 2, *P *= 0.401; day 3, *P *= 0.055; day 4, *P *= 0.64; day 5, *P *= 0.805).

**Table 3:** Number of interventions for intracranial hypertension

	Mean serum sodium			*P *value
		
	< 145 mEq/l	145 to 155 mEq/l	> 155 mEq/l	
Day 1	4.35 ± 2.33	4.31 ± 2.02	2.00 ± 0	0.354
Day 2	4.16 ± 2.73	2.67 ± 1.39	3.00 ± 0	0.033
Day 3	5.12 ± 3.97	2.91 ± 1.82	3.17 ± 3.49	0.018
Day 4	3.60 ± 3.50	3.41 ± 2.59	2.33 ± 2.00	0.527
Day 5	3.19 ± 2.44	1.84 ± 0.90	2.67 ± 1.53	0.043
Total	4.20 ± 2.91	2.95 ± 1.97	2.62 ± 2.25	< 0.001

Data presented as mean ± standard deviation number of interventions per day, providing a summary of the mean number of interventions for intracranial pressure control on days 1 to 5. Patients were divided into three groups based on their mean serum sodium value on each day of the study period. For each day, statistical comparisons were performed for the number of interventions required in each group.

**Figure 3:**
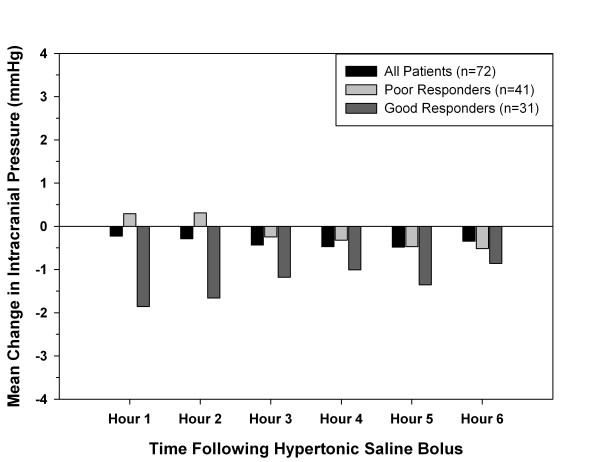
**Mean change in intracranial pressure for 6 hours following a hypertonic saline bolus**. Poor responders demonstrated a positive response to 0 to 49% of hypertonic saline boluses. Good responders demonstrated a positive response to 50 to 100% of hypertonic saline boluses.

The acute effect of HTS on ICP was evaluated for 322 HTS boluses in 72 patients. Overall, the mean ± standard deviation ICP did not significantly change in the 6 hours following a HTS bolus (baseline, 13.7 ± 8.4 mmHg; hour 1, 13.6 ± 8.3 mmHg; hour 2, 13.5 ± 8.8 mmHg; hour 3, 13.3 ± 8.7 mmHg; hour 4, 13.4 ± 8.7 mmHg; hour 5, 13.4 ± 8.3 mmHg; hour 6, 13.5 ± 8.3 mmHg; *P *= 0.84). When poor responders were analyzed separately, no significant change was again noted (baseline, 12.5 ± 6.1 mmHg; hour 1, 13.2 ± 6.6 mmHg; hour 2, 13 ± 6.7 mmHg; hour 3, 12.6 ± 5.8 mmHg; hour 4, 12.5 ± 5.6 mmHg; hour 5, 12.6 ± 6.1 mmHg; hour 6, 12.5 ± 5.7 mmHg; *P *= 0.16). Good responders demonstrated a statistical trend toward lower ICP values following a HTS bolus (baseline, 16.1 ± 11.2 mmHg; hour 1, 14.3 ± 10.8 mmHg; hour 2, 14.3 ± 11.7 mmHg; hour 3, 14.8 ± 12.3 mmHg; hour 4, 15.1 ± 12.5 mmHg; hour 5, 15 ± 11.3 mmHg; hour 6, 15.4 ± 11.4 mmHg; *P *= 0.068). Figure 3 shows the change in ICP following a HTS bolus for all patients, poor responders and good responders. Demographics for patients in the poor and good responder groups were not different except for the median (interquartile range) Injury Severity Score (poor responders, 30 (25 to 38); good responders, 38 (29 to 43); *P *= 0.029).

## Discussion

### Effects of targeting mild hypernatremia

The current study is the largest to evaluate the correlation of serum sodium and ICP in patients with TBI. These retrospective results did not demonstrate a correlation between serum sodium and ICP. This finding was consistent when patient data were analyzed using multiple different methods and for multiple subgroups. The administration of HTS did not consistently result in increases in serum sodium as seen in Figure 2. This would suggest a reason for the lack of significant changes in ICP. It is unknown whether a more substantial increase in serum sodium would result in further decreases in ICP. Based on Figure 2, even serum sodium increases of > 2 mEq/l did not consistently result in a decrease in ICP.

Statistically, there were fewer mean interventions for ICP control in patients with mean serum sodium > 145 mEq/l. However, there was only a statistical difference on three of the 5 days studied. This finding is not clinically significant, as it is expected that patients with serum sodium < 145 mEq/l would receive more HTS doses in an attempt to achieve the goal for serum sodium (145 to 155 mEq/l). In fact, when HTS doses were excluded from the analyses, no difference in the number of interventions among groups was seen at any point in the study period. An alternative explanation is that serum sodium > 145 mEq/l truly did have a beneficial effect in decreasing the number of interventions. Overall, we feel that this is unlikely because there was no correlation between ICP and serum sodium in any of the analyses.

The poor correlation between ICP and serum sodium found in the current study is perplexing when compared with data from Qureshi and colleagues. They retrospectively evaluated the use of continuous infusion 3% saline to target a serum sodium between 145 and 155 mEq/l, and they found that increased serum sodium concentrations correlated with lower ICP in the eight patients with TBI (*R*^2 ^= 0.91, *P *= 0.03) and five with postoperative edema (*R*^2 ^= 0.82, *P *= 0.06) [6]. Overall, the mean ICP was reduced by 6.9 ± 2.4 mmHg, suggesting that continuous infusion of HTS could reduce ICP in a select group of patients. Although baseline ICP values were not specifically defined, the mean ICP reported prior to HTS administration (14.2 ± 4.2 mmHg) is lower than the mean baseline ICP of 19.1 ± 11.5 mmHg seen in the current study. Additionally, the current study evaluated significantly more patients with TBI and the majority of patients received HTS boluses (84%) rather than continuous infusions.

Roquilly and colleagues retrospectively investigated the use of 20% HTS in 50 TBI patients. They used a continuous infusion to target serum sodium of 145 to 155 mEq/l [10]. Mean ICP significantly decreased by the first hour from 31 ± 9 to 21 ± 8 mmHg (*P *< 0.05). Mean serum sodium significantly increased within the first 4 hours from 140 ± 4 to 144 ± 4 mEq/l (*P *< 0.05). Although no statistical evaluation was performed to correlate serum sodium with ICP, it appears that ICP decreased as serum sodium increased. While this study focused on TBI, the treatment of these patients was different from that in the current study due to the fact that they used HTS as a third-line agent after mannitol and sodium thiopental by continuous infusion, while the current study used HTS as the first-line agent.

Conversely, data from Shackford and colleagues support the current study. They prospectively compared the use of continuous infusion of 1.6% HTS (*n *= 18) versus lactated Ringer's solution (*n *= 16) in patients with TBI [11]. There was no difference in mean ICP between groups, and the number of daily interventions for ICP management was higher on days 1 and 5 for patients who received HTS. The authors attributed these findings to a trend for lower admission GCS scores in the HTS group. Similarly, Qureshi and colleagues evaluated the use of continuous infusion of 2 to 3% saline (*n *= 36) versus normal saline (*n *= 48) in patients with TBI [12]. In-hospital mortality was higher in patients who received HTS (odds ratio, 3.1; 95% confidence interval, 1.1 to 10.2), despite no difference in admission GCS scores or the number of interventions for ICP management between groups.

Overall, the four previous studies in this area and the current study show mixed results. Two studies suggested that increasing the serum sodium improved ICP control (total *n *= 58) and two did not (total *n *= 113). The fifth study suggested that continuous infusion of HTS may be detrimental. As such, these results call into question the targeting of mild hypernatremia for ICP control.

### Acute effects of hypertonic saline on intracranial pressure

The more commonly reported use of HTS in TBI has been for acute treatment of elevated ICP. Regarding the acute effects of HTS boluses in the current study, this therapy did not result in statistically significant reductions in ICP overall. However, there was a group of patients that seemed to respond. These findings are important given that poor ICP control is associated with increased mortality and effective pharmacologic management is a key part of therapy [13,14]. The results of this study are significant because using HTS for management of intracranial hypertension is not yet clearly defined in the Brain Trauma Foundation guidelines [2].

The data are also mixed for bolus administration of HTS. Several studies reported an acute decrease in ICP by 5 to 15 mmHg using HTS boluses [4,5,15-19]. In the current study there was a less profound effect with no significant change in the ICP following a HTS bolus. However, the good responder group did demonstrate a reduction in ICP that approached significance (*P *= 0.068). It is important to note that the methods between studies have not been uniform and patients have varied by type and severity of illness. The best method of HTS administration has obviously yet to be determined. This is especially true in light of studies that have highlighted the dangers of hypernatremia from continuous infusions of HTS, including an increased risk of death in ICU patients [20-22].

### Strengths and limitations

The current study has strengths and limitations. The retrospective design and the lack of a control group are the most significant limitations. Owing to the current standard of care in our institution, it was not possible to find a control group that did not receive HTS. The inability to relate the time of neurosurgical interventions is problematic due to the effect such procedures could have on ICP. Unfortunately, the retrospective nature of the study only allowed for collection of whether or not a patient underwent a procedure. However, collection of these data was consistent with the American Brain Injury Consortium methods used in clinical trials. Another limitation relates to the documentation of ICP. Each ICP value is simply a snapshot over the course of each hour. Also, hospital policy allowed nurses to have a 1-hour time window to administer a HTS dose relative to the documented time in the medication administration record. These are pragmatic issues inherent to any retrospective study in this field.

The primary strength of the study is the large sample size (*n *= 81). This is the largest study to date examining the use of HTS in this fashion. Another strength of the study was the relatively homogeneous patient population, including only patients with TBI. Finally, while there was not a formal treatment protocol for administration of HTS, there was a relatively uniform approach to treatment of TBI by the neurosurgeons in the ICU.

## Conclusion

Serum sodium values of critically ill patients with TBI do not correlate with ICP - although patients with serum sodium values < 145 mEq/l required more interventions for intracranial hypertension. Patients who had a good acute response to HTS boluses trended toward a decrease in ICP following each dose compared with poor responders. Targeting a serum sodium value of 145 to 155 mEq/l requires further evaluation.

## Key messages

• Serum sodium values do not correlate well with ICP in patients with TBI treated with HTS.

• Achieving serum sodium values between 145 and 155 mEq/l may decrease the number of daily interventions required to manage elevated ICP.

• HTS boluses did not significantly decrease ICP overall.

## Abbreviations

GCS: Glasgow Coma Scale; HTS: hypertonic saline; ICP: intracranial pressure; NMB: neuromuscular blocker; TBI: traumatic brain injury.

## Competing interests

The authors declare that they have no competing interests.

## Authors' contributions

DLW, GCW, BAB, and JMS designed the study. DLW, CGH, and LJM collected clinical data. DLW analyzed the raw data, performed statistical analyses, and drafted the manuscript. JMS advised on the performance of statistical analyses. DLW, GCW, BAB, JMS, CGH, LJM, MAC, MSM, and TCF participated in the interpretation of all data and in manuscript revisions, and provided clinical and scientific guidance regarding the content of the manuscript. All authors read and approved the final manuscript.
